# Risk Factors and Clinical Outcomes of Arterial Re-Occlusion After Successful Mechanical Thrombectomy for Emergent Intracranial Large Vessel Occlusion

**DOI:** 10.3390/jcm13247640

**Published:** 2024-12-15

**Authors:** In-Hyoung Lee, Sung-Kon Ha, Dong-Jun Lim, Jong-Il Choi

**Affiliations:** Department of Neurosurgery, Korea University Ansan Hospital, Korea University College of Medicine, 123 Jeokgeum-ro, Danwon-gu, Ansan 15355, Gyeonggi-do, Republic of Korea; hiphopdick@naver.com (I.-H.L.); sungkonha@gmail.com (S.-K.H.); djlim@korea.ac.kr (D.-J.L.)

**Keywords:** acute ischemic stroke, re-occlusion, risk factors, thrombectomy

## Abstract

**Background:** Re-occlusion of initially recanalized arteries after thrombectomy is a significant concern that may lead to poor outcomes. This study aimed to identify the risk factors and evaluate the prognosis of arterial re-occlusion following successful thrombectomy in patients diagnosed with emergent large-vessel occlusion (ELVO). **Methods:** We retrospectively analyzed data from 155 consecutive patients with ELVO who underwent mechanical thrombectomy (MT). Patients were classified into two groups according to whether the initial recanalized artery was re-occluded within 7 days after successful thrombectomy: re-occlusion and non-occlusion groups. Multivariate analysis was performed for potentially associated variables with *p* < 0.20 in the univariate analysis to identify the independent risk factors of re-occlusion. Differences in clinical outcomes were also assessed in these two groups. **Results:** Re-occlusion occurred in 10.3% of patients (16/155). Multivariate analysis demonstrated that large artery atherosclerosis (odds ratio [OR]: 3.942, 95% confidence interval [CI]: 1.247–12.464; *p* = 0.020), the number of device passes (OR: 2.509, 95% CI: 1.352–4.654; *p* = 0.004), and residual thrombus/stenosis (OR: 4.123, 95% CI: 1.267–13.415; *p* = 0.019) were independently associated with re-occlusion. Patients with re-occlusion had significantly worse NIHSS scores at discharge and lower opportunities for achieving functional independence at 3 months after MT than patients without re-occlusion. **Conclusions:** Large artery atherosclerosis, a high number of thrombectomy device passes, and residual thrombus/stenosis seemed to promote re-occlusion after successful recanalization. Timely identification and proper treatment strategies to prevent re-occlusion are warranted to improve clinical outcomes, especially among high-risk patients.

## 1. Introduction

Mechanical thrombectomy (MT) is currently considered the standard of care for treating acute ischemic stroke secondary to emergent large-vessel occlusion (ELVO), and several randomized clinical trials have demonstrated its efficacy [1,2,3]. With the improvement of endovascular techniques, devices, and accumulated user experience, the successful recanalization rate has increased to >80% in individuals with ELVO [4,5,6,7]. However, it remains a significant concern that some patients may suffer re-occlusion of the initial recanalized vessel within hours or days. In previous studies, the prevalence of re-occlusion was reported in approximately 5–10% of patients with ELVO after successful thrombectomy [8,9,10,11]. Additionally, re-occlusion may impose neurological deterioration and lead to an unfavorable prognosis [10,12]. Consequently, it is necessary to determine the factors associated with re-occlusion in real-world clinical practice and adjust the relevant modifiable factors to improve clinical outcomes. Unfortunately, available data regarding the associated factors for re-occlusion are limited, and previous studies report conflicting results regarding its risk factors [9,12,13,14,15].

Therefore, we aimed to identify the risk factors associated with postprocedural arterial re-occlusion after successful thrombectomy in patients diagnosed with anterior circulation ELVO by comparing the clinical and radiological characteristics between patients with and without re-occlusion. Additionally, we sought to evaluate the clinical outcomes of re-occlusion in these cohorts.

## 2. Materials and Methods

### 2.1. Study Design

All consecutive patients with anterior circulation ELVO who received MT at our institution between January 2016 and March 2024 were retrospectively reviewed. We only included patients who had successful recanalization on the final cerebral angiography. Furthermore, we restricted this investigation to cerebral-angiography-established occlusion of the intracranial internal carotid artery and proximal middle cerebral artery (MCA-M1). We excluded patients who lacked available angiographic and radiological data.

**Figure 1:**
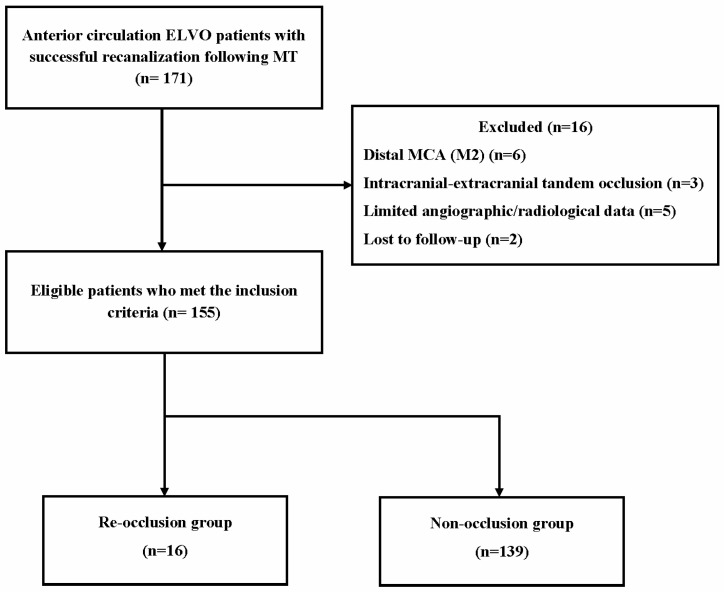
Flowchart of the patient selection. ELVO, emergent large vessel occlusion; MT, mechanical thrombectomy; MCA, middle cerebral artery.

Patients were dichotomized into two groups based on whether the initial recanalized artery was re-occluded within 7 days after successful thrombectomy: re-occlusion and non-occlusion groups. Figure 1 shows the flowchart for this study’s patient selection process. Our institution’s ethical committee approved the current study; owing to its retrospective nature, written informed consent was waived.

### 2.2. Data Collection

The following radiological and clinical data were acquired and analyzed: demographics, medical history, baseline National Institutes of Health Stroke Scale (NIHSS) score, stroke etiology determined according to the Trial of Org. 10172 in Acute Stroke Treatment (TOAST) criteria [16], initial Alberta Stroke Program Early CT Score (ASPECTS) based on brain computed tomography (CT) scan, and arterial occlusion site. The following procedure-related variables were also retrieved and evaluated: intravenous thrombolysis pretreatment, onset-to-puncture time, procedure time, use of balloon guiding and aspiration catheters, number of device passes, and permanent intracranial stent insertion.

### 2.3. Thrombectomy Procedure and Medication

Stroke-trained neurologists performed intravenous thrombolysis on eligible patients before the procedure in accordance with the current guidelines [17]. All thrombectomy procedures were performed by dual-trained neurovascular surgeons. A balloon guide catheter (mainly Cello; Medtronic) was preferred; however, this was determined according to the preference of the attending surgeons. The type of stent most commonly used was a Solitaire FR (Medtronic), which is capable of retrieval and detachment and can be used both as a first-line device for MT and for permanent stenting, if necessary. The number of retrieval attempts was not restricted and was left to the operator’s discretion. Cerebral angiography was used to evaluate the recanalization status following each device pass. When non-occluding residual embolic fragments or vessel wall stenosis were identified following successful recanalization on final cerebral angiography, permanent stent insertion was considered at the operator’s discretion. If the successfully treated artery was revealed to be re-occluded, repeated thrombectomy was performed using the same technique. Additionally, we administered an intra-arterial glycoprotein IIb/IIIa inhibitor (tirofiban; the total dose ranged from 0.5 mg to 1.0 mg) [14] and/or rescue stenting at the discretion of the neurovascular surgeons. A loading dose of 300 mg aspirin was administered orally or through a nasogastric tube for patients who underwent permanent stenting. After the procedure, dual antithrombic medications (100 mg aspirin and 75 mg clopidogrel) were provided to all patients in whom hemorrhagic complications were ruled out on brain CT.

### 2.4. Angiographic and Clinical Outcomes

**Figure 2:**
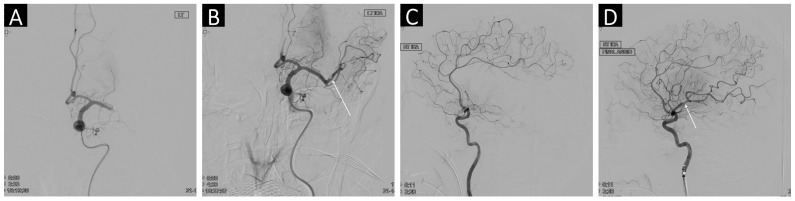
Illustrative images of two patients who underwent successful recanalization with residual nonocclusive thrombus or stenosis. (**A**) Angiographic imaging showed a left M1 occlusion. (**B**) The final angiographic image after recanalization disclosed an intraluminal filling defect, suggesting residual nonocclusive thrombus (white arrow). (**C**) The right middle cerebral artery was occluded in the M1 segment. (**D**) The final angiography demonstrated successful recanalization with focal stenosis (white arrow).

The modified Thrombolysis in Cerebral Infarction (mTICI) score was used to evaluate angiographic results. A final cerebral angiography demonstrating an mTICI score >2b was deemed successful recanalization [18]. The presence of remaining embolic fragments or stenosis presenting intraluminal focal filling defects after successful recanalization on final cerebral angiography was further analyzed (Figure 2) [12]. Re-occlusion of the treated vessel after successful thrombectomy was defined as recurrent occlusion of the same target artery where a thrombectomy was performed [19]. The re-occlusion was confirmed by follow-up vascular imaging (mainly magnetic resonance imaging with angiography) conducted 7 days after the procedure, or earlier in cases of neurological worsening. Occlusion of the other large vessel or iatrogenic embolization in previously unaffected regions during the procedure was not regarded as re-occlusion [10]. Angiographic data were evaluated by two physicians blinded to clinical information. Clinical outcomes included the NIHSS scores at discharge, functional outcomes, mortality, and the occurrence of hemorrhagic complications. Functional outcomes were assessed 3 months after treatment using the modified Rankin Scale (mRS) scores. We defined mRS scores ≤2 as functional independence. Intracranial hemorrhage (ICH) detected on postprocedural brain CT scans or follow-up magnetic resonance imaging was regarded as symptomatic ICH if the NIHSS score increased by >4 points from baseline [20].

### 2.5. Statistical Analysis

Comparisons between the re-occlusion and non-occlusion groups were performed using Student’s *t*-test or Mann–Whitney U test to analyze continuous variables. The results were provided as means with standard deviations or medians with interquartile ranges. Categorical variables, presented as frequencies, were analyzed using Pearson’s Chi-squared statistic or Fisher’s exact test, as appropriate. Variables with a *p*-value of <0.2 in the univariate analysis were entered into a backward stepwise likelihood ratio multivariate binary logistic regression model as candidate variables. Multivariate binary logistic regression analysis assessed each variable’s contribution to the postprocedural re-occlusion of the treated vessel. We tested our logistic regression models for predictive accuracy using Nagelkerkes *R*^2^. The odds ratio (OR) with its 95% confidence interval (95% CI) was estimated for each variable. A *p*-value of <0.05 was considered statistically significant. All statistical analyses were performed using the SPSS 25.0 software (IBM, Armonk, NY, USA).

## 3. Results

### 3.1. Baseline Characteristics

The current study enrolled 155 consecutive patients with ELVO who satisfied the inclusion criteria (mean age: 69.9 ± 12.2 years; 52.3% female and 47.7% male). Among the entire cohort, re-occlusion occurred in 16 patients (10.3%) within 7 days after successful recanalization. The remaining 139 patients were classified to the non-occlusion group (Figure 1). The clinical and demographic variables of patients with and without re-occlusion are compared in Table 1. The two groups had comparable demographical characteristics, except for the stroke etiology. More patients were revealed to have underlying large artery atherosclerosis etiology according to the TOAST classification in the re-occlusion compared to the non-occlusion group (50.0% vs. 23.7%; *p* = 0.024). Regarding procedure-related variables, patients with re-occlusion had a significantly higher number of device passes than those without (2.4 ± 1.3 vs. 1.8 ± 0.7; *p* = 0.004). Moreover, residual thrombus or stenosis was more frequently detected on final cerebral angiography in the re-occlusion group compared with the non-occlusion group (43.8% vs. 19.4%; *p* = 0.026).

### 3.2. Risk Factors for Re-Occlusion

On univariate analysis, a history of diabetes mellitus (*p* = 0.133), antiplatelet pretreatment (*p* = 0.156), underlying large artery atherosclerosis etiology (*p* = 0.024), number of device passes (*p* = 0.004), and residual thrombus/stenosis (*p* = 0.026) were identified as potential independently associated factors for re-occlusion after successful thrombectomy (*p* < 0.2); therefore, these factors were included in the multivariate analysis. Multivariate analysis demonstrated that large artery atherosclerosis (OR: 3.942, 95% CI: 1.247–12.464; *p* = 0.020), the number of device passes (OR: 2.509, 95% CI: 1.352–4.654; *p* = 0.004), and residual thrombus/stenosis (OR: 4.123, 95% CI: 1.267–13.415; *p* = 0.019) were independently associated with re-occlusion. However, diabetes mellitus and antiplatelet pretreatment were not significantly correlated with re-occlusion (*p* > 0.05; Table 2).

### 3.3. Clinical Outcomes

The disparities in clinical outcomes between both groups are summarized in Table 3. The discharge NIHSS score was significantly higher in patients with than without re-occlusion (15 [9.75 to 22] vs. 8 [4 to 12]; *p* < 0.001). Furthermore, 3-month mRS scores were significantly higher in the re-occlusion group than in the non-occlusion group (3 [2 to 5] vs. 2 [1 to 3]; *p* = 0.017). Overall, functional independence was achieved in 56.8% (88/155) of patients. The re-occlusion group achieved a significantly lower rate of functional independence than the non-occlusion group (31.3% vs. 59.7%; *p* = 0.037). The incidence of symptomatic ICH and mortality were comparable between the two groups (*p* > 0.05).

## 4. Discussion

Arterial re-occlusion occurred in 10.3% (16/155) of patients with anterior circulation ELVO after initial successful thrombectomy. The present study identified several risk factors for re-occlusion despite successful recanalization: an underlying etiology of large artery atherosclerosis, a high number of thrombectomy device passes, and residual thrombus/stenosis on final cerebral angiography. Furthermore, re-occlusion corresponded with increased NIHSS scores at discharge and a poor functional prognosis at 3 months. The likelihood of functional independence in patients undergoing re-occlusion was reduced by approximately half, supporting the importance of arterial patency in achieving a good clinical outcome.

The prevalence of arterial re-occlusion following successful endovascular revascularization is not well established, and currently few related studies exist due to its relatively low incidence. According to our results, roughly 10% of patients suffer arterial re-occlusion. This is slightly higher than the 6.59% (126/1883) reported in a recent meta-analysis [8]; however, there is a vast variation in incidence (ranging from 2.3 to 29.5%) owing to considerable heterogeneity in the timing of assessment and diagnostic imaging modalities for re-occlusion across studies. Hence, a unified definition of postprocedural re-occlusion is warranted.

Our results demonstrate that underlying large artery atherosclerosis as a suspected stroke etiology was significantly related to re-occlusion. This concurs with a previous study demonstrating that re-occlusion was influenced by an atherosclerotic etiology [10,12]. Moreover, another study demonstrated that atherosclerosis-related stroke has a relatively poorer outcome than that of embolic stroke, attributable to the higher rate of re-occlusion [21]. However, the etiology of ELVO is often discovered just a few days or even weeks after stroke onset, making it difficult to determine the cause of the stroke during its acute phase [19]. Therefore, careful assessment of imaging or angiographic surrogate markers that suggest atherosclerosis-related stroke before or during the procedure should be emphasized to help operators establish a treatment strategy to prevent re-occlusion [22].

Intracranial atherosclerotic stenosis is generally recognized as a therapeutic challenge with a high risk of instant or delayed re-occlusion despite the comparable rate of recanalization success with embolic stroke [23,24]. Therefore, several studies have suggested alternative treatment strategies (stent implantation with or without angioplasty) for intracranial atherosclerosis-related ELVO [25,26]. The protective effect of permanent stenting on re-occlusion was reported in a recent retrospective study [9]. In our cohorts, re-occlusion occurred in only 6.7% (2 of 30) of patients who underwent permanent stenting. While our results did not demonstrate statistical significance between stenting and re-occlusion, possibly owing to the small sample size, this finding may serve as informative support for future research on treatment strategies to prevent re-occlusion in patients diagnosed with intracranial atherosclerosis-related ELVO.

Consistent with previous studies reporting that a higher number of device passes increases the risk of re-occlusion after successful thrombectomy, our results revealed an independent association between the number of thrombectomy attempts and re-occlusion [9,10,11]. Multiple thrombectomy device passes contribute to re-occlusion through the following theoretical mechanisms: (1) multiple device passes may cause vessel injury at the target artery, which may lead to re-occlusion resulting from vasospasm and dissection; (2) repeated manipulations of a microwire and microcatheter may provoke accumulated arterial endothelial injury [25,27,28]. Additionally, there is consensus on the detrimental effects of multiple thrombectomy attempts on clinical outcomes [29]; thus, it can be assumed that re-occlusion due to multiple device passes may have contributed to worsening clinical outcomes.

In line with a previous study reporting that 25% of patients with residual stenosis or thrombus fragments at the target artery experienced re-occlusion, the current study found a re-occlusion rate of 20.6% in similar cases [12]. The remaining thrombus fragments can serve as a nidus to which the higher concentration of circulating platelets may have adhered, resulting in re-occlusion [10]. Residual arterial stenosis is typically observed in cases with underlying atherosclerosis and is more likely to be caused by an unstable plaque leading to re-occlusion. Unfortunately, there may be cases where residual stenosis or thrombus was not recognized on the final angiography because the distal flow was not hindered if successful reperfusion was achieved. A delayed angiographic run and cautious angiographic assessment at the end of the procedure are recommended to prevent missing residual debris or an underlying plaque, which may result in re-occlusion, especially in high-risk patients. Appropriate identification of residual stenosis or thrombus may provide the opportunity to perform additional endovascular procedures (e.g., repeat thrombectomy, permanent stenting, intra-arterial tirofiban infusion) in a timely manner to improve clinical outcomes [14,26].

The results of the current study should be interpreted cautiously, considering several limitations. First, our findings might have been inherently constrained by the retrospective, single-center design, and our analysis might have lacked statistical power owing to the relatively small patient population (particularly in the re-occlusion group). During the patient selection process, we excluded patients who were unable to undergo follow-up vascular imaging for a variety of reasons, including life-threatening medical complications, early mortality, or the severity of their stroke. This may have led to underestimations of the proportion of patients with re-occlusion, considering that patients with more severe clinical presentations were more likely to have experienced re-occlusion. Additionally, we failed to evaluate the following potentially associated variables that have been documented to contribute to the re-occlusion: platelet count [12], D-dimer level [9], and previous statin pretreatment [10]. Further studies designed with larger, multicenter cohorts are necessary to validate our results and improve the identification of patients diagnosed with anterior circulation ELVO at risk of re-occlusion following successful thrombectomy.

## 5. Conclusions

The current study revealed that a stroke etiology of large artery atherosclerosis, a high number of thrombectomy device passes, and residual thrombus/stenosis on final cerebral angiography may promote re-occlusion after successful recanalization, corresponding with the patient’s poor functional prognosis. These results highlight the necessity of timely identification and proper treatment strategies to prevent re-occlusion, especially in patients considered at high risk of suffering it.

## Figures and Tables

**Table 1:** Baseline characteristics.

	Re-Occlusion (*n* = 16)	Non-Occlusion (*n* = 139)	*p*-Value
Sex, male	8 (47.1)	66 (47.5)	0.855
Age, years, mean (SD)	69.3 ± 14.4	69.9 ± 11.9	0.857
Medical history			
Hypertension	7 (43.8)	66 (47.5)	0.785
Diabetes mellitus	7 (43.8)	36 (25.9)	0.133 *
Dyslipidemia	5 (31.3)	44 (31.7)	0.975
Current smoking	4 (25.0)	36 (25.9)	0.940
Coronary heart disease	4 (25.0)	33 (23.7)	0.916
Atrial fibrillation	3 (18.8)	43 (30.9)	0.315
Chronic kidney disease	3 (18.8)	19 (13.7)	0.634
Anticoagulant pretreatment	3 (18.8)	24 (17.3)	0.890
Antiplatelet pretreatment	3 (18.8)	51 (36.7)	0.156 *
Baseline NIHSS score, median (IQR)	17.5 (11.5–21.5)	17 (11–20)	0.801
ASPECTS, median (IQR)	8 (7–9)	8 (8–9)	0.706
Site of occlusion			0.208
Intracranial ICA	8 (50.0)	45 (32.4)	
MCA - M1	8 (50.0)	94 (67.6)	
Stroke etiology			
Large-artery atherosclerosis	8 (50.0)	33 (23.7)	0.024 *
Cardioembolic	8 (50.0)	94 (67.6)	0.208
Other or unknown	0 (0)	8 (5.8)	0.328
Procedure-related data			
IVT	9 (56.3)	95 (68.3)	0.379
Use of balloon guiding catheter	10 (62.5)	86 (61.9)	0.962
Use of aspiration catheter	4 (25.0)	36 (25.9)	0.940
Time metrics			
Onset-to-puncture, min, mean (SD)	274.4 ± 41.4	262.7 ± 54.6	0.316
Onset-to-IVT, min, mean (SD)	176.7 ± 25.6	167.4 ± 47.7	0.566
IVT-to-puncture, min, mean (SD)	98.3 ± 17.3	87.3 ± 28.6	0.258
Procedure time, min, mean (SD)	51.6 ± 13.3	47.8 ± 17.8	0.318
Number of device passes, mean (SD)	2.4 ± 1.3	1.8 ± 0.7	0.004 *
Number of device passes > 2	6 (37.5)	23 (16.5)	0.046
Residual thrombus or stenosis	7 (43.8)	27 (19.4)	0.026 *
Permanent stenting	2 (12.5)	28 (20.1)	0.416

Data are presented as numbers (percentages) unless otherwise indicated. * Included in the multivariate analysis. SD, standard deviation; NIHSS, National Institutes of Health Stroke Scale; IQR, interquartile range; ASPECTS, Alberta Stroke Program Early CT Score; ICA, internal carotid artery; MCA, middle cerebral artery; IVT, intravenous thrombolysis.

**Table 2:** Multivariate analysis for factors associated with re-occlusion.

Variables	Adjusted OR (95% CI)	*p*-Value
Diabetes mellitus	2.465 (0.708–8.585)	0.156
Antiplatelet pretreatment	0.463 (0.112–1.916)	0.288
Large-artery atherosclerosis	3.942 (1.247–12.464)	0.020 *
Number of device passes	2.509 (1.352–4.654)	0.004 *
Residual thrombus or stenosis	4.123 (1.267–13.415)	0.019 *

Nagelkerkes *R*^2^: 0.367. * Statistically significant OR, odds ratio; CI, confidence interval.

**Table 3:** Comparison of clinical outcomes.

	Re-Occlusion (*n* = 16)	Non-Occlusion (*n* = 139)	*p*-Value
NIHSS scores at discharge, median (IQR)	15 (9.75–22)	8 (4–12)	<0.001 *
mRS scores at 3 months, median (IQR)	3 (2–5)	2 (1–3)	0.017 *
Functional independence	5 (31.3)	83 (59.7)	0.037 *
Mortality	3 (18.8)	14 (10.1)	0.296
Any ICH	5 (31.3)	38 (27.3)	0.759
Symptomatic ICH	2 (12.5)	10 (7.2)	0.555

Data are presented as numbers (percentages) unless otherwise indicated. * Statistically significant NIHSS, National Institutes of Health Stroke Scale; IQR, interquartile range; mRS, modified Rankin Scale; ICH, intracranial hemorrhage.

## Data Availability

The datasets generated during and/or analyzed during the current study are available from the corresponding author on reasonable request.
